# Management and Prevention Strategies for Non-communicable Diseases (NCDs) and Their Risk Factors

**DOI:** 10.3389/fpubh.2020.574111

**Published:** 2020-11-26

**Authors:** Aida Budreviciute, Samar Damiati, Dana Khdr Sabir, Kamil Onder, Peter Schuller-Goetzburg, Gediminas Plakys, Agne Katileviciute, Samir Khoja, Rimantas Kodzius

**Affiliations:** ^1^Panevezys Faculty of Technology and Business, Kaunas Technology University (KTU), Panevezys, Lithuania; ^2^Department of Biochemistry, Faculty of Science, King Abdulaziz University (KAU), Jeddah, Saudi Arabia; ^3^Division of Protein Science, Kungliga Tekniska Högskolan Royal Institute of Technology, Stockholm, Sweden; ^4^Department of Medical Laboratory Science, Charmo University, Chamchamal, Iraq; ^5^Procomcure Biotech, GmbH, Thalgau, Austria; ^6^Prosthetics, Biomechanics and Biomaterial Research, Paracelsus Medical University Salzburg, Salzburg, Austria; ^7^Bioprospecting Departament, Baltic Institute of Advanced Technology, Vilnius, Lithuania; ^8^Faculty of Medicine, Ludwig Maximilian University of Munich (LMU), Munich, Germany

**Keywords:** risk factors, non-communicable diseases, health policy, prevention strategies, healthcare-management

## Abstract

Non-communicable diseases (NCDs) are of increasing concern for society and national governments, as well as globally due to their high mortality rate. The main risk factors of NCDs can be classified into the categories of self-management, genetic factors, environmental factors, factors of medical conditions, and socio-demographic factors. The main focus is on the elements of self-management and to reach a consensus about the influence of food on risk management and actions toward the prevention of NCDs at all stages of life. Nutrition interventions are essential in managing the risk of NCDs. As they are of the utmost importance, this review highlights NCDs and their risk factors and outlines several common prevention strategies. We foresee that the best prevention management strategy will include individual (lifestyle management), societal (awareness management), national (health policy decisions), and global (health strategy) elements, with target actions, such as multi-sectoral partnership, knowledge and information management, and innovations. The most effective preventative strategy is the one that leads to changes in lifestyle with respect to diet, physical activities, cessation of smoking, and the control of metabolic disorders.

## Introduction

**Figure 1:**
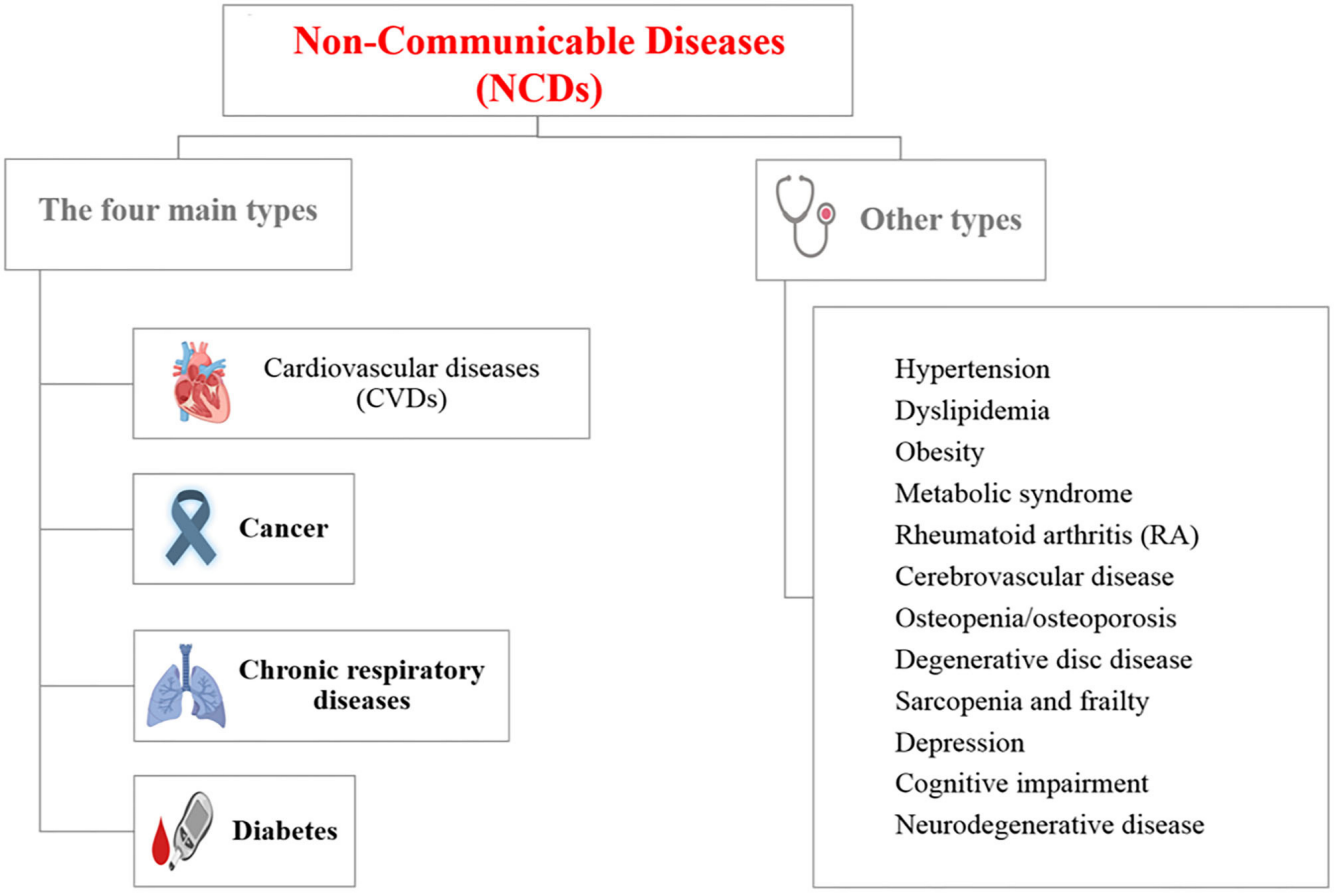
List of non-communicable diseases (NCDs) [Created with BioRender].

Non-communicable diseases (NCDs), also known as chronic diseases, are medical conditions that are associated with long durations and slow progress (Figure 1). Most NCDs are non-infectious and are the result of several factors, including genetic, physiological, behavioral, and environmental factors (1). According to the World Health Organization (WHO), NCDs are the leading cause of death worldwide, responsible for 71% of the total number of deaths each year. The top four killers among NCDs with the highest number of deaths are cardiovascular diseases (17.9 million deaths annually), cancers (9.0 million), respiratory diseases (3.9 million), and diabetes (1.6 million) (Figure 1) (1). However, the term of NCDs has been extended to cover a wide range of health problems, such as hepatic, renal, and gastroenterological diseases, endocrine, hematological, and neurological disorders, dermatological conditions, genetic disorders, trauma, mental disorders, and disabilities (e.g., blindness and deafness) (2). The main risk factors contributing to NCDs involve unhealthy diets, physical inactivity, tobacco use, and alcohol misuse. Hence, most of these diseases are preventable as they eventually progress in early life due to lifestyle aspects (3). There is an increasing concern that poor diet has increased the potential risk, causing chronic diseases, and nutrition problems in the public health sector (4). Historically, many NCDs have been directly linked to economic growth and were called “diseases of the rich.” Now, the burden of NCDs in developing countries has increased. Further, mortality in low and middle-income countries has doubled the burden of NCDs. The growing interest in population well-being and economic growth, based on Gross National Happiness (GNH), has recently attracted more attention. The epidemic of NCDs hinders the progress of GNH because good health is necessary in order to achieve happiness (5). Bhutan's experience suggests that strategic opportunities to minimize NCDs and to promote population well-being can be taken advantage of by joining the health sector with other sectors at the individual and organizational levels (5).

Health and well-being are the primary goals of society in regards to food choice (6). Researchers have pointed out that the core of the health-conscious lifestyle is directed toward a wellness-oriented lifestyle (5) and the behavior of people determines their health status (7). Nutritionists have been reported to be associated with many chronic diseases, but designed studies exploring the association between diet, nutrition, and NCDs are rare (8). Thus, lifestyle modifications and interventions to reduce the risk of NCDs is the priority in the primary prevention of diseases. Hence, finding answers to the following questions can significantly contribute to a better and healthier society:

- What are NCDs and their risk factors?
- What are the most used interventions in managing the risk of NCDs?
- What are the contemporary prevention strategies for NCDs?

The current review focuses on the answers to the previous questions and highlights several strategic models in the contemporary management of NCDs.

## Key Risk Factors of NCDs

**Figure 2:**
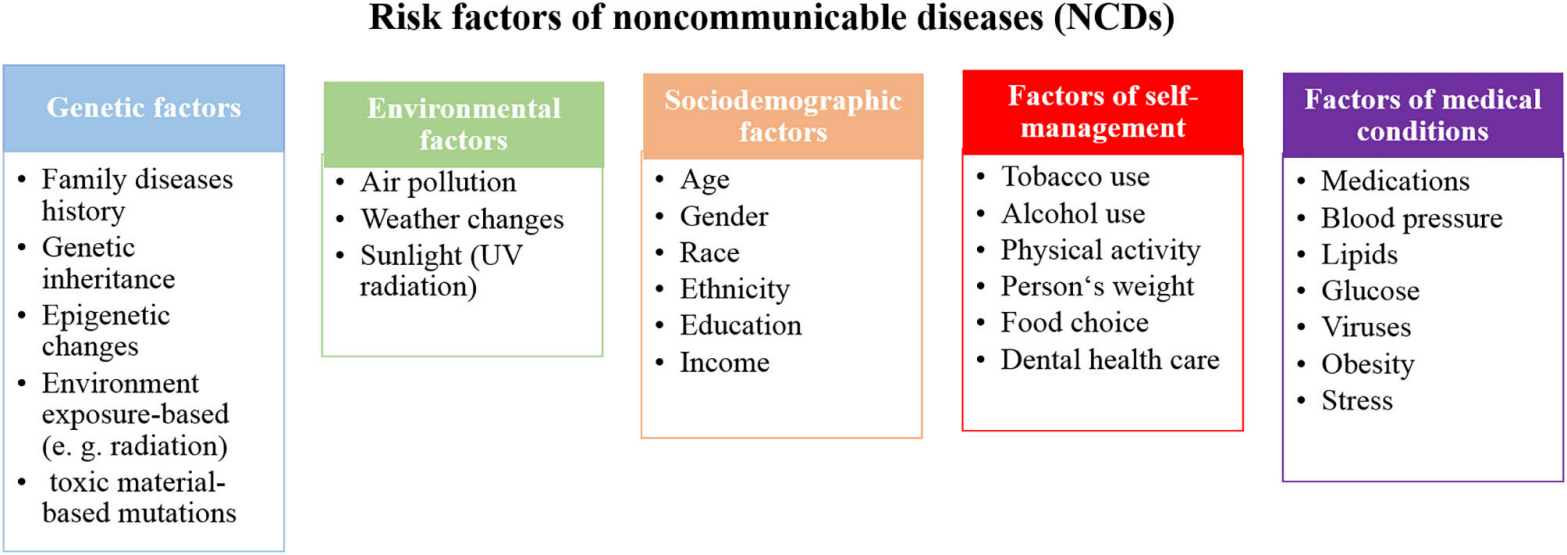
A proposed model to classify the risk factors of NCDs.

Several factors can increase the amount of opportunities to develop NCDs and can be classified in different ways. In one approach, risk factors are classified as modifiable or non-modifiable factors that can have changeable or non-changeable conditions, respectively. The modifiable risk factors involve high blood pressure, smoking, diabetes mellitus, physical inactivity, obesity, and high blood cholesterol, while the non-modifiable risk factors involve age, gender, genetic factors, race, and ethnicity (9–12). Interestingly, although age and gender are non-modifiable factors, most of their associated factors are modifiable. Figure 2 represents a model to classify the risk factors of NCDs. The non-modifiable factors can also be classified into three classes: (i) biological factors, such as being overweight, dyslipidemia, hyper-insulinaemia, and hypertension; (ii) behavioral factors, such as diet, lack physical activity, tobacco smoking, and alcohol consumption; and (iii) societal factors, which involve complex combinations of interacting socioeconomic, cultural and environmental parameters (13). In the next section, examples of the identified risk factors for NCDs, including age, diet, and economic context, are highlighted.

### Age

While NCDs are usually associated with elderly people, all ages are at risk, even before birth. These diseases may start in the earliest years of life and keep progressing during childhood, adolescence, and old age (14). However, 15 million deaths due to NCDs were recorded from people aged between 30 and 69 years of age and more than 82% of these “premature” deaths were from low and middle-income countries (15). The life-course perspective is evidence of the origin of adult NCDs, which are determined in uterus. Barker (16) showed that maternal nutrition plays a significant role in adult diseases. He found that adapting human fetuses to a limited supply of nutrients resulted in permanent structure and metabolism changes. Subsequently, such programmed changes may have attributed to several diseases, such as heart disease, diabetes and hypertension in later life (16, 17). Moreover, unborn babies are not only negatively influenced by maternal habits, such as diet, drug, stress, alcohol and tobacco consumption during pregnancy, but environmental factors, such as air pollution, also have an effect. These factors influence the fetal and early brain development, for example, a low birth weight is attributable to poor long-term health and poor cognition (14, 18).

In the period of childhood, new risks of NCDs may appear due to the easy access to unhealthy food and drinks in kindergartens and schools. Thus, this leads to a high number of overweight and obese children (19). After that stage of life, young people in the adolescence stage can acquire new and harmful habits, such as smoking and drinking alcohol, which can significantly contribute to NCD risk (20, 21). These bad habits may continue during adulthood with additional aspects facing adults in workplaces, including financial stressors, unemployment, unsatisfying careers, and low social engagement, which influence the progress of NCDs (20, 22). Retirement and leaving a workplace can provide new challenges among elderly people and influence the development of NCDs. Poor nutrition, lack of physical activity, alcohol and tobacco use, social isolation, and financial stress directly affect older people and strongly promotes NCDs (20).

The prevention and control of NCDs can be achieved at all ages. The health status of women before and during pregnancy influences the susceptibility of children to NCDs in later life (20, 23). This is the most important strategy to control NCDs because it targets the root of the problem. Applying high standards for food and drinks, increased physical activity in schools and workplaces, in addition to monitoring air quality and offering smoke-free zones can largely prevent NCDs at all stages of life. However, taxation and creating restricted policy for the marketing of unhealthy food, sugary drinks, tobacco, and alcohol can largely improve health statistics. Further, as obese children and elderly people are at a high risk of social isolation, it is important, for their mental and physical health, to be involved in social activities (20, 24).

### Diets and Lifestyle

In the past, infectious and parasitic diseases were the main causes of death, but in the recent decades, NCDs have replaced them and have become the main cause of deaths (25). This may be attributed to the change of diet habits and lifestyle over the years, which can be classified as a shift of disease patterns in humans. Various dietary factors, such as meat, whole grain products, healthy dietary patterns, sugar-sweetened beverage consumption, and iron-based diets have an obvious relationship with NCDs (11, 12). Additionally, the high consumption of processed meat and sugar-sweetened beverages, combined with other unhealthy lifestyle factors, such as a high body mass index (BMI), physical inactivity, and smoking have a marked association with NCDs (26, 27). Whole-grain products are independent of the BMI and have protective effects, due to their high fiber contents and ability to slowly release glucose into circulation; subsequently, this reduces the postprandial insulin response and may improve insulin sensitivity (26, 28–31).

Dietary transition describes the changes in production, processing, availability, dietary consumption, and energy expenditure. Further, the concept becomes wider and involves body composition, anthropometrical parameters, and physical activity (32, 33). The use of dietary transition terms arises due to the shift to western diets in developing countries in particular. Traditional food in most countries is healthier, natural, and richer in fiber, and cereal has been replaced by unhealthy processed food that is rich in sugars and fats, animal-source foods, and refined carbohydrates. Hence, low and middle-income countries have seen rapid changes in nutrition transition and rapid increases in NCDs (34). High food consumption and declining physical activity rates occur simultaneously, resulting in NCDs. The main factor, attributable to physical inactivity, is the rapid and continuous development in technology. The easy access to modern technology and manufacturing in houses and workplaces, including machines, vehicles and labor-saving technology, make life easier but unhealthier from the perspective of reducing the risk of NCDs (34).

### The Economic Context

NCDs are already common in developed countries and rapidly propagate. Spreading western lifestyle in low and middle-income countries, due to global population aging and commercial pressures for unhealthy diets and cigarettes, contributes to the increasing rate of NCDs in these countries (35). There is a direct relationship between poor health and low-income, which contributes to food poverty, purchasing of cheaper and unhealthy dietary products, and expensive treatments, in addition to psychosocial factors. People with low-incomes have the feeling that they occupy a lower status in society, which prevents them from participating in social life (36). However, food poverty, poor mobility and lack of physical activity are also serious problems in high-income countries (37).

There is a growing trend to consider social, political, and economic systems as critical factors that impact NCDs besides individual behavior/lifestyle (38, 39). Krieger's Ecosocial theory highlights ecosocial disease distribution which describes how diversity between historical, societal, and ecological conditions significantly contributes to changes in the health outcomes of various social groups (39). For example, the bad side of economic and health inequality that already exists for many years becomes obvious with the current coronavirus COVID-19 pandemic. According to Krieger's research, the higher number of COVID-19 deaths in African American than whites in the US is attributed to several factors involve living in crowded places, using public transportation to commute to work, working in service jobs in close contact with others, and shortage of protective equipment at workplaces. Furthermore, the lack of access to health care and health insurance, and pre-existing health conditions may be increased the risk from COVID-19 in the African American population (40).

## Key Diseases

### Cardiovascular Diseases (CVDs)

CVDs are the leading contributors to the global burden of disease among the NCDs and account for the most deaths worldwide each year—even more than the number of deaths from cancer and chronic respiratory diseases combined (41, 42). CVDs are a group of disorders that are not only related to heart conditions, such as ischaemic heart disease (IHD), stroke, congenital heart disease, coronary heart disease, cerebrovascular disease, peripheral arterial disease, and rheumatic heart disease, but also to blood vessels that involve hypertension, and conditions associated with cerebral, carotid, and peripheral circulation (43). While CVDs equally affect both sexes, men suffer from higher incidences than women. Still, CVDs are the leading cause of death of women in developed countries (44). Moreover, many epidemiological studies show the relationship between periodontal disease (PD) and cardiovascular disease. Mild forms of PD affect 75% of adults in the US, and more severe forms affect 20 to 30% of adults. Since PD is common, it is responsible for a significant proportion of proposed infection-associated risks of cardiovascular diseases (45, 46).

According to the American Heart Association, there are seven key health factors and behaviors that contribute to the increasing risks of heart disease and stroke: nutrition, smoking, overweight/obesity, physical inactivity, uncontrolled blood pressure, elevated levels of cholesterol, and blood sugar (42).

Most CVDs can be prevented by addressing the seven risk factors, which involves healthy diets, regular physical activity, avoiding smoking and second-hand smoking, reaching and maintaining a healthy weight, and keeping blood pressure, cholesterol, and blood sugar levels under control (42).

### Cancer

Cancer is the main public health problem and the second main cause of death globally [who]. It shares the common risk factors with other key diseases of NCDs and several identified and unidentified factors can be attributed to cancer. The causes of cancer can be classified into three categories, including: (i) biological carcinogens (e.g., viral, bacterial, or parasites infections, hormonal and genetics factors); (ii) chemical carcinogens (such as food and water contamination, and tobacco smoking); and (iii) physical carcinogens (such as ultraviolet and ionizing radiation). However, tobacco smoking is considered to be the main cause of cancer, followed by poor diets (47–49). Moreover, together, body weight and lack physical activity are also associated with the most common cancers, including breast (postmenopausal), colon, endometrium, kidney, and esophagus cancers (50). According to WHO report in 2018, the most common cancers are lung, breast, colorectal, prostate, skin, and stomach, while the most cancer deaths are from cancer of the lung, colorectal, stomach, liver, and breast (48). A noticeable decrease in the cancer death rates of lung, breast, colon/rectum, and prostate is achieved in high-income countries, but are still high in low and middle-income countries (51). Further, the incidence of several cancers, including lung, breast, prostate, colon, and rectum, is commonly elevated concurrently with economic development. In contrast, the incidence of stomach cancer declines with economic development (48). The guidelines for oncological disease prevention and early detection are based on cancer risk assessment, including past medical history, lifestyle factors, family diseases history, and genetic testing (10).

Lung cancer, which is the most common cancer in the world, is mainly the result of smoking and the risk increases in heavy smokers (52). Further, several studies reported low intakes of fruits, vegetables and related nutrients in lung cancer patients (53, 54). Hence, it is possible to prevent lung cancer by stopping the prevalence of smoking and by increasing fruit and vegetable consumption. Furthermore, dietary habits and physical activity contribute to breast cancer, which is the second most common cancer in the world and the most common cancer among women. Excess adiposity and hormonal mechanisms appeared to play key roles in breast cancer progress, and are effected by dietary intake during childhood and adolescence (51, 55, 56). Hence, maintaining a healthy weight throughout life can minimize the chances of breast cancer. Another type of cancer that is strongly associated with diet is colorectal cancer. High intakes of meat and fat, and low intakes of fruits and vegetables, dietary fiber, vitamins and minerals are related to an increased risk of colorectal cancer (57). Hence, minimizing or stopping the consumption of meat, especially preserved meats, can reduces the risk of this cancer. Stomach cancer was the main cause of mortality globally, but is currently decreasing in industrialized countries. It is associated with dietary habits and vitamin C intake (48). *Helicobacter pylori* infection is considered to be a type I carcinogen and as the strongest known risk factor of gastric cancer (58). Cancers caused by infections are three times lower in developed countries than in developing ones. It is important to avoid the infection in order to prevent cancer, and that can be achieved by eating food that is properly prepared, drinking water from clean sources, taking vitamins according to the recommended dietary allowance, and avoiding the extensive use of antibiotics in order to reduce antibiotic resistant strains (51).

### Chronic Respiratory Diseases (CRDs)

CRDs cover a wide range of diseases in the airways and the other structures of the lungs. Most of the morbidity and mortality of CRDs is increased with age. CRDs include chronic obstructive pulmonary disease (COPD), occupational lung diseases, asthma and respiratory allergies, sleep apnoea syndrome, and pulmonary hypertension. Asthma and COPD account for most of the deaths among CRDs in low and middle-income countries (59–61). Genetic and environmental factors are the risk factors of CRDs; environmental factors are more dominant. These factors include air pollution exposure, including tobacco smoke and second-hand tobacco smoke, indoor and outdoor air pollution, occupational exposures, and socioeconomic factors (62, 63).

CRDs are not fully reversible and are partially preventable (64). During pregnancy, maternal smoking contributes to lung dysfunction in children at birth. Further, in early life, a child's health affects their subsequent respiratory health. Thus, following a healthy lifestyle in the early ages of life, avoiding respiratory infections, and avoiding environmental and occupational agents can effectively prevent CRDs. Preventing exposure to indoor and outdoor pollutants can be achieved by filtration and ventilation, in addition to the use of natural gas (27).

### Diabetes Mellitus

Diabetes has attracted global attention due to its elevating prevalence and incidence. It is not only a chronic disease, but also an acutely life-threatening condition. Further, it may cause other serious diseases such as heart diseases, kidney failure, and eye damage, which may subsequently lead to blindness, and foot ulcers, which may require limb amputation. The main two types of diabetes are both lead to hyperglycemia. In type 1, the pancreatic β-cells cannot produce a sufficient amount of insulin, while in type 2, the body cells cannot respond properly to insulin (64). Other types of diabetes involve gestational diabetes mellitus, which occurs in pregnant women with glucose intolerance (65), and type 3 diabetes, which is associated with Alzheimer's disease, where neurons in the brain cannot respond to insulin (66). While diabetes can be partially inherited, several lifestyle factors, such as obesity, high sugar consumption, and lack of physical activity can significantly contribute to the progress diabetes. However, lifestyle changes can prevent diabetes and the long-term complications of diabetes. Patients with type 2 diabetes can control or even reverse the diabetes by changing their lifestyle and eating habits. The term “healthy dietary pattern” includes a variety of diets and nutritional factors, for example, reducing the consumption of red and processed meat, sugar-sweetened beverages and alcohol, while increasing the consumption of whole-grain products (67).

## Management of Risk Factors and NCDs

The following sections outline the developed and proposed strategies to manage NCDs and their risk factors from several perspectives.

### Management of Risk Factors

The most common causes of NCDs are metabolic and behavioral risk factors and can be largely preventable by several available means. Most global discussions concern the risk factors of self–management (tobacco and alcohol consumption, physical activity, weight, food, and dental health care) and focus on the role of individual responsibility to manage the risk factors of NCDs. Health care specialists should educate patients about their nutrition value and raise the profile of didactics, practicums, and workshops in daily practice (68). Further, the management of NCDs is the priority of the public health sector in most countries, because management in society is the main direction of NCD prevention strategies. Interventions are used in public health management in an effort to promote good health behavior. For example, India, with its wide sociocultural, economic, and geographical diversity, is implementing multi-sectoral (partnership between different sectors) actions to prevent NCDs, including school health programs, initiatives of National Cancer Control Programme, National Trauma Control program, National Program for Control of Blindness, National Mental Health Programme, the National Tobacco Control Program, and the National Program for Control of Diabetes, Stroke, and Cardiovascular Diseases initiatives (69). From another approach, researchers also highlight the environmental factors (air pollution, climate changes, sunlight) and their impact on NCD development. Air pollution will be an important challenge in the future and new technologies, such as microchips, will have more of an impact in air monitoring (27).

Since diet is a common risk factor among most NCDs, it attracts more attention in an effort to find effective strategies to provide healthy food to the community and at all stages of life. Evidence-based nutrition interventions should be a global health priority and the role of the dietary fat studied should be a modifiable variable in the prevention of NCDs (29). Recent evidence suggests that a diet that is high in healthy fat and rich in unsaturated fatty acids prevents the development of metabolic diseases and reduces cardiovascular events (29). Many interventions addressing poverty and development have an impact on NCD prevalence and risk (69). The current evidence is limited to diets, and a positive effect of agricultural-based food security programs on diet indicators has been suggested (7). A suboptimal diet is the leading risk factor for NCDs and consumption of specific foods, rather than macronutrients or micronutrients; it may be the most significant risk factor for NCDs (70). Strategic health communication in the population-wide intervention includes engaging the food industry in order to reduce the salt content in foods (71). The concept of a sustainable diet combines health and environmental concerns and includes the abovementioned risk factors as part of the recommendations to reduce processed meat consumption and to increase whole-grain consumption (72). Lifestyle activities include healthy diets and focus on limiting the use of salt, sugar, and saturated fats (73). While our body can synthesize many of the molecules required to function properly, essential nutrients are obtained from food. Carbohydrates, proteins, and fats are the primary components of food. Minerals are inorganic essential nutrients that must be obtained from food. The omega−3 alpha-linolenic and the omega−6 linoleic acids are essential fatty acids that are needed to make some membrane phospholipids. Vitamins (B, C, A, D, E, and K) are the classes of essential organic molecules (such as cofactors) that are required in small quantities for most enzymes to function properly. The absence or low levels of vitamins can have a dramatic effect on health. A focus on the need to meet adequate dietary intakes of essential nutrients (74) through a healthy diet is considered to be very significant for the aging society (74). Food supplements are concentrated sources of nutrients (minerals and vitamins) or other substances with a nutritional or physiological effect, which are marketed in the form of pills, capsules, and/or liquids (Table 1). These dietary supplements offer many benefits, including the maintaining of an adequate intake of certain nutrients, to correct nutritional deficiencies, or to support specific physiological functions. Recently, researchers have been looking for new solutions to implement an efficient food production process and to discover the benefits of starch waste on human health.

**Table 1:** Types of food supplements.

**Type of food supplements**	**Role**	**Examples**
Vitamins (75, 76)	organic chemical compounds that low or high intake level can cause certain diseases or symptoms	- Water-soluble vitamins: C, B_1_, B_2_, B_5_, B_6_, B_9_, B_12_ - Fat-soluble vitamins: A, D, E, K
Dietary minerals (77–79)	chemical elements indispensable for life. Some minerals are essential for the enzymes to function properly	K, Cl, N, Ca, P, Mg, Fe, Zn, Mn, Cu, I, Se, Co
Proteins and amino acids (80, 81)	Amino acids are the building blocks of proteins, classified into two classes: essential cannot be synthesized in the body and required in the diet and non-essential that synthesized in the body.	- Essential: histidine, isoleucine, leucine, lysine, methionine, phenylalanine, valine - Non-essential: Alanine, serine, asparagine, aspartic acid, glutamic acid
Bodybuilding supplements (81–83)	Dietary supplements used by people involved in bodybuilding, weightlifting, and athletics	food products with high protein contents, essential fatty acids, weight loss products
Essential fatty acids (84, 85)	Nutrients cannot be synthesized in the body and required in the diet	Alpha-linolenic acid (omega-3 fatty acid), linoleic acid (omega-6 fatty acid)
Natural products (86, 87)	Extract from plants, animals, algae, fungi, or lichens	Ginkgo biloba, curcumin, cranberry
Probiotics (88, 89)	Living microorganisms (bacteria, yeast) that orally consumed to offer benefits for the digestive system	*Leuconostocmesenteroides, Lactobacillus plantarum, Pediococcuspentosaceus, Lactobacillus brevis, Leuconostoccitreum, Leuconostocargentinum, Lactobacillus paraplantarum, Lactobacillus coryniformis, Bifidobacterium bifidum, Streptococcus thermophilus*

### Management of NCDs

NCDs are the silent killers threatening health without showing any symptoms until the problem progresses to an advanced stage. Patients with NCDs, or people with a susceptibility to develop one, need long-term care that is personalized, proactive, and sustainable. Primary health care can organize and deliver healthcare strategies to manage NCDs in each community and to detect diseases at early stages. Thus, they can significantly overcome the challenges linked to a high cost in the health sector. For example, several studies have proved that lifestyle factors have direct links to cancer risk and changing lifestyles, in a positive approach, can considerably minimize the cancer burden. The main risk factors of cancer are age, gender, alcohol, smoking, family disease history, and food (90–92). Cancer can be prevented by changing behavior: dietary improvements, physical activity, weight control, obesity management, tobacco prevention, safe sex and control of oncogenic viruses, sun protection, medications, and lower alcohol consumption (26).

A dramatic decrease in all cardiovascular disease-related deaths has been recorded in high-income countries, whereas a significant increase was registered in low and middle-income countries (93, 94). Checkley et al. reported on NCDs' management in low and middle-income countries (95). While some people in these countries can access the same treatments that are available in high-income countries, the majority of the population lacks access. The main obstacle causes an increase in the number of patients with NCDs in low and middle-income communities is the absence of a well-designed plan to stop disease occurrence and spreading. Each country needs to prepare its management plan, not just with coping models from high-income countries. Several successful models have been verified, taking into consideration the low-cost strategies to prevent, diagnose and treat NCDs. For example, a cost-effective strategy has been developed in Kenya to diagnose diabetes and hypertension in the early stages of life. While health workers are visiting homes to examine human immunodeficiency virus (HIV) infection, they also measure blood glucose levels and blood pressure. Further, type 2 diabetes is a global pandemic that highly affects human health and global economic development (96). The International Diabetes Federation reported that there were 415 million people living with type 2 diabetes in 2015, and estimated that the number by 2040 might increase to 642 million, which is attributable to genetic and environmental factors (96). The genetic–environmental interaction induces insulin resistance and β-cell dysfunction (96). The epidemic of type 2 diabetes in recent decades has not only attributed to the alteration of the gene pool, but environmental changes also play significant roles in the rapid increase in the prevalence of type 2 diabetes (96). However, global diabetes mellitus epidemics require the looking for innovative approaches to prevention (7).

### Contemporary Prevention Strategy of NCDs

**Figure 3:**
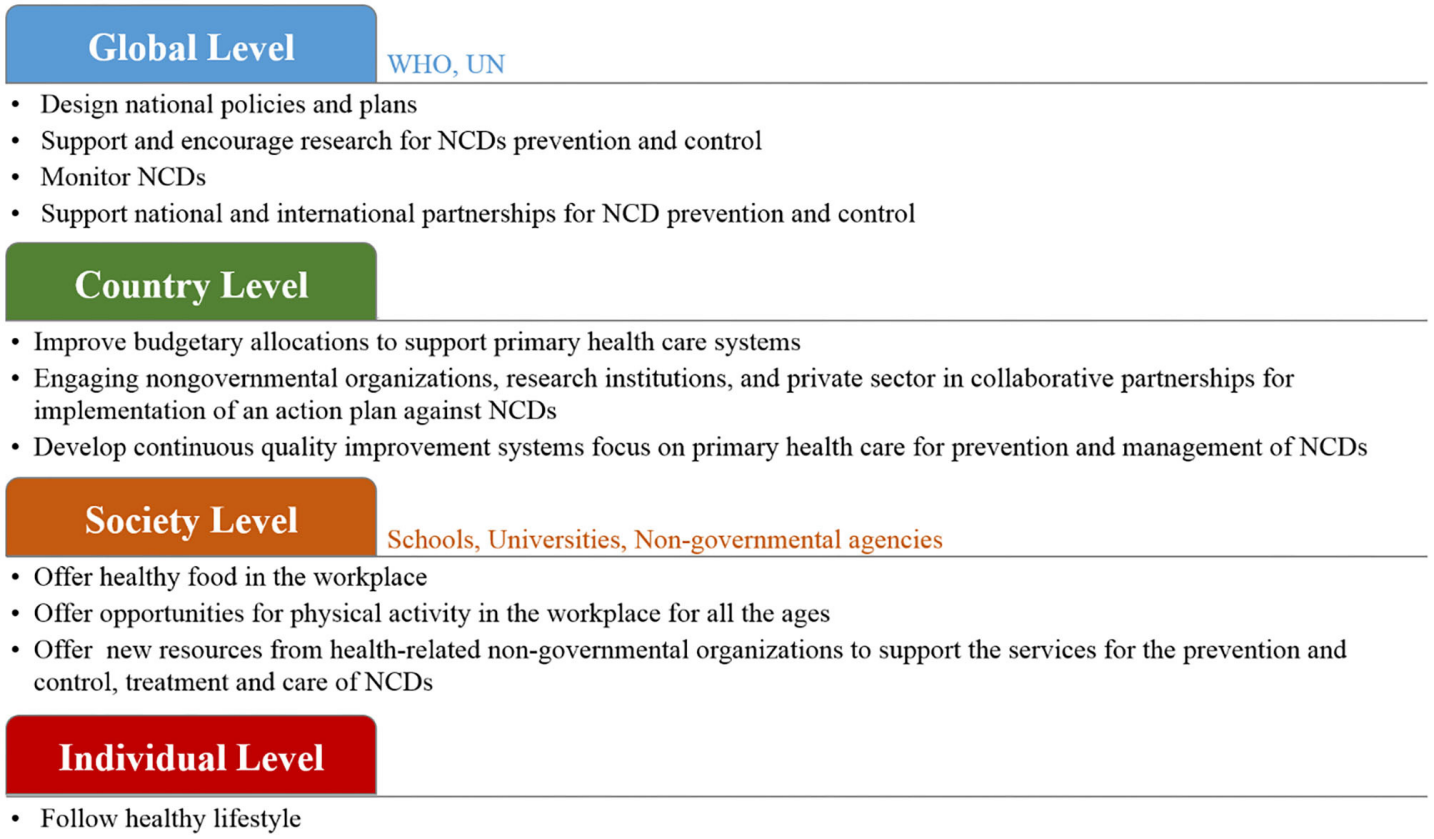
The proposed prevention management of NCDs with small and large-scale human cooperation.

The prevention strategies of NCDs can include small and large-scale human cooperation (Figure 3). The importance of preventing NCDs arises from the direct impact of NCDs on the decreasing rate of national income. Loss productivity on a large-scale is the result of the inability to work and the frequent absence threats to the national economy. The management strategy to prevent NCDs is based on risk factor management that addresses individual, society, country, and global levels, with actions, such as resource allocation, multi-sectoral partnership, knowledge and information management and innovations. The most critical dimension of the prevention strategy is lifestyle management at the individual level and with a focus on actions, such innovations, which can help the society to increase the awareness of risk factors management, to take health policy decisions at a country level and to develop a health strategy at the global level. The importance of leadership for the change management process is underscored and requires the creation of new approaches to the prevention of NCDs (96, 97).

At the global level, WHO and UN agencies can work together to design policies and strategies to reduce the risk of NCDs (98, 99). It is important to monitor NCDs and to assess their progress at the national, regional and global levels. These organization can support research and encourage collaborations among national and international health agencies and academic institutions. Further, tobacco smoke, as a common factor of the four main types of NCDs, must be put under control. The WHO offers help to smokers who have the desire to stop using tobacco products and to implement rules to propose a smoke-free environment. Further, WHO can, by law, protect tobacco control policies from the commercial interests of the tobacco industry. At the country level, each government needs to design its plan based on its economy. Several low-cost and highly effective strategies are available to prevent and manage NCDs (100–103). For example, encouraging people to play sports for physical activity is the most effective factor that can easily influence the prevention of NCDs, and at the same it is time and cost-effective. Moreover, improved budgetary allocations to support primary health care systems should be put in place in order to provide health services to all community members. To achieve large-scale progress, collaboration between governments and various non-governmental organizations, schools, and universities, to provide advice on lifestyle modifications and to warn people about the risks of NCDs, is in high demand. At the society level, research centers and institutes can significantly contribute to the prevention of NCDs by conducting research projects and programs. Focusing research on food biotechnology and agriculture has a direct influence on NCDs risk (7, 104). The development of diagnostic tools allows for the rapid detection of NCDs biomarkers with high sensitivity to help detect diseases at their early stages, which subsequently contributes to easier treatment and fast cures (105–107). However, in order to reach the highest attainable standard of health, it is important to encourage individuals and families to follow a healthy lifestyle in order to get an effective response for prevention and the control of NCDs and to improve health outcomes (100, 108).

## Conclusions

In modern society, NCDs are the main challenge in health systems. Risk factor management is essential in NCDs' management. The management of NCDs requires many strategies from several perspectives and on different levels, including the individual and country levels. Based on the hypotheses that were raised during the above scientific discussion, it can be concluded that modern strategies for the management of NCDs should be oriented toward the individual level, where the individual is responsible for their health by simply following a healthy lifestyle. It is important to combine modern scientific achievements and innovative decisions, with regard to the rationality of nutrition and positive effects on human health. Governments and international organizations should make people aware of their health and their environment to make the world a safe and healthy place. From another perspective, support research to find new techniques to improve food biotechnology is in high demand. Further, finding rapid and sensitive diagnostic platforms to detect NCDs at the point-of-care offers huge benefits to personnel and the healthcare system. The innovations are vital to address the growing crisis of NCDs successfully, and most often use lifestyle projects, the promotion of healthy eating behaviors and smoking cessation. We believe that there is a need to look for further innovations to build better lives in society.

## Author Contributions

The manuscript was prepared by AB, SD, and RK. Writing review and editing was done by AB, SD, DS, KO, PS-G, GP, AK, SK, and RK. Final revision and approval was done by RK. All authors contributed to the article and approved the submitted version.

## Conflict of Interest

KO was employed by the company Procomcure Biotech, GmbH. The remaining authors declare that the research was conducted in the absence of any commercial or financial relationships that could be construed as a potential conflict of interest.
